# Phosphorus stocks and flows in an intensive livestock dominated food system

**DOI:** 10.1016/j.resconrec.2020.105065

**Published:** 2020-12

**Authors:** S.A. Rothwell, D.G. Doody, C. Johnston, K.J. Forber, O. Cencic, H. Rechberger, P.J.A. Withers

**Affiliations:** a Lancaster Environment Centre, Lancaster University, Lancaster, UK; b Agri Food and Biosciences Institute, Belfast, Northern Ireland, UK; c Institute for Water Quality and Resource Management, TU Wien, Vienna, Austria

**Keywords:** Phosphorus, Substance flow analysis, Food system, Livestock, Northern Ireland

## Abstract

Current use and management of phosphorus (P) in our food systems is considered unsustainable and considerable improvements in the efficiency of P use are required to mitigate the environmental impact of poor P stewardship. The inherent low P use efficiency of food production from animals means food systems dominated by livestock agriculture can pose unique challenges for improving P management. This paper presents the results of a substance flow analysis for P in the Northern Ireland (NI) food system for the year 2017 as a case study for examining P stewardship in a livestock dominated agricultural system. Imported livestock feed was by far the largest flow of P into the NI food system in 2017 (11,700 *t* ± 1300 t) and P from livestock excreta the largest internal flow of P (20,400 ± 1900t). The P contained in livestock slurries and manures alone that were returned to agricultural land exceeded total crop and grass P requirement by 20% and were the largest contributor to an annual excess soil P accumulation of 8.5 ± 1.4 kg ha^−1^. This current livestock driven P surplus also limits the opportunities for P circularity and reuse from other sectors within the food system, e.g. wastewater biosolids and products from food processing waste. Management of livestock P demand (livestock numbers, feed P content) or technological advancements that facilitate the processing and subsequent export of slurries and manures are therefore needed.

## Graphical Abstract

**Figure:**
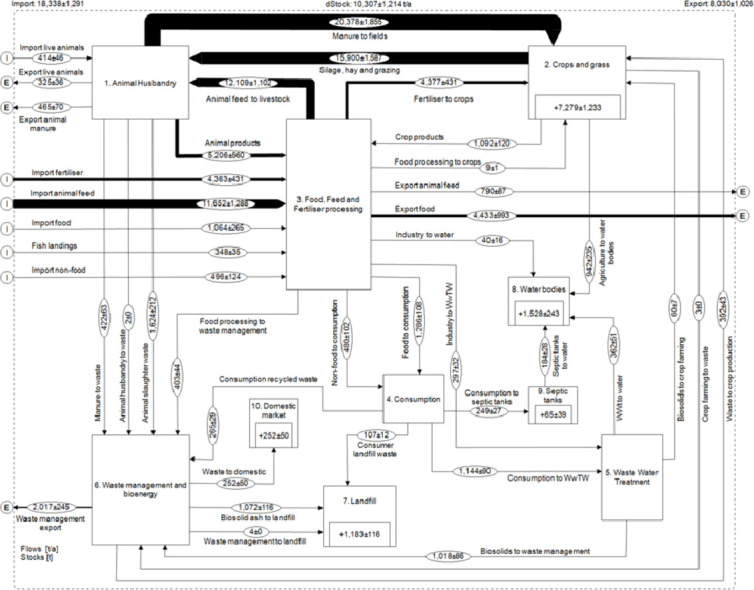

AD: Anaerobic Digestion
AHDB: Agriculture and Horticulture Development Board
CV: Coefficient of Variation
DAERA: Department of Agriculture, Environment and Rural Affairs
DCW: Dressed Carcass Weight
DEFRA: Department for Environment, Food and Rural Affairs
EU: European Union; NI Northern Ireland
NIEA: Northern Ireland Environment Agency
P: Phosphorus
RAE: Relative Agronomic Efficiency
SFA: Substance Flow Analysis
SRP: Soluble Reactive Phosphorus
STAN: subSTance flow ANalysis
WFD: Water Framework Directive
WwTW: Wastewater Treatment Works

## 1 Introduction

The unsustainability of current phosphorus (P) use is now well recognised (Sharpley et al., 2018). While it is an essential nutrient for global agriculture and food production (Dawson and Hilton, 2011) its inappropriate management in the food chain causes widespread environmental degradation of our fresh and coastal waters (Mekonnen and Hoekstra, 2018) and global P flows are considered to be outside the ‘safe operating space’ of planetary boundaries for maintaining the earth system (Steffen et al., 2015). Furthermore, the unbalanced global distribution of known mineable phosphate rock reserves means that future mineral P fertiliser supplies will likely be subject to increasing geopolitical and economic pressures (Elser et al., 2014). Better stewardship of the P needed for our food systems is more important than ever to safeguard future food and water security. Sustainable management of P in our food system should involve moving away from current linear models of P use, minimising losses into waste management storage such as landfill, and increasing the recycling and thus circularity of P, consequently reducing reliance on P imports, particularly mineral P fertilisers (Nesme and Withers, 2016). Reducing P losses to the environment is also critical in helping mitigate the ecologically damaging impact of P on our water resources (Smith, 2003).

While agriculture is only one component of a food system, it often dominates the P stocks and flows within it. Sustainable management of P in livestock dominated food systems poses a unique set of challenges due to a combination of factors *inter alia*, reliance on animal feed concentrates, distances to arable farming areas, cost of storing and transporting manures, land requirements for application of manures, competition for land resources with other organic manures, matching crop P requirement with organic P availability and use of inorganic fertilisers due to a lack of trust in organic P fertilizer (Bateman et al., 2011; Hooda et al., 2000; Liu et al., 2017; Nicholson et al., 2012). These challenges result in many livestock areas operating at regional, farm and/or field scale P surpluses, causing a build-up of P in soil and untimely slurry applications, both of which increase P losses to waterbodies, with consequences for biological and chemical water quality (Hooda et al., 2000; Leip et al., 2015; Senthilkumar et al., 2012). While surplus P is negative in terms of environmentally sustainable agriculture, it can potentially reduce the vulnerability of these areas to shocks related to global P availability by providing a buffer against inorganic P fertiliser shortages and price fluctuations. However, the knock-on impact of such P shocks on grain producing areas would have significant consequences for livestock areas, particularly those heavily reliant on imported feeds.

Whilst it is acknowledged that increased circularisation and minimisation of P losses in the food system are crucial steps toward increasing P sustainability and reducing its vulnerability to P shocks, there are additional challenges to improving food system P efficiency, including minimising P demand and matching actual P requirements in different parts of the system with appropriate supply (Withers et al., 2018). This requires connectivity between the different sectors that make up a food system. To realistically improve the sustainability of P management within any food system, a thorough understanding of how P moves through those systems is first required.

Substance flow analysis (SFA) is an approach often adopted to both analyse and visualise P movement within agricultural and food systems, allowing identification and quantification of imports, exports, accumulations and losses of P within a geographically defined system. SFA has been previously used to describe P flows at regional (Chowdhury et al., 2018; Hanserud et al., 2016; Senthilkumar et al., 2012), national (Cooper and Carliell-Marquet, 2013; Cordell et al., 2013; Klinglmair et al., 2015) and global (Liu et al., 2008; Vaccari et al., 2019) scales and is often considered a starting point for improved P stewardship by highlighting priority areas for intervention to meet future sustainability targets for P stewardship (Brunner, 2010).

The aim of this study was to create a SFA for elemental P in a livestock dominated food system, using Northern Ireland (NI) as a case study to help identify the challenges and opportunities in achieving circularisation and minimisation of P at the regional scale.

## 2 Methods

### 2.1 Study area

Similar to the rest of Europe, NI has no phosphate rock reserves and is thus reliant on P imports, predominantly as animal feed and fertiliser, to satisfy its food system P demand. Agriculture in NI is dominated by livestock farming, with 95% of agricultural land under grassland including rough grazing. In 2018, livestock output of meat, milk and eggs represented 87% of gross agricultural output (DAERA, 2018b). However, similar to other livestock intensive agricultural systems such as those found in Denmark (Klinglmair et al., 2015), Belgium (Coppens et al., 2016) and the Netherlands (Van Middelkoop et al., 2012), historic over application of P-rich animal slurries and manures have resulted in excessive soil P levels that are well above crop requirement. A recent national soil sampling scheme showed that 38% of all agricultural soils in NI have levels of crop-available P that are above the agronomic optimum recommended for grassland, and within the dairy sector 50% are above optimum (Higgins et al., 2020).

**Fig. 1:**
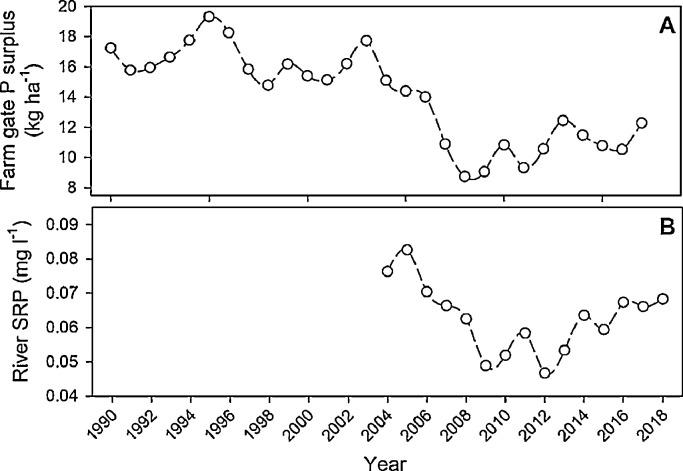
(A) Farmgate agricultural phosphorus surplus in Northern Ireland from 1990 to 2017 (data published from permission from DAERA) and (B) monitored average annual riverine soluble reactive phosphorus (SRP) from 2004 to 2018 (data published with permission from NIEA).

In 2017, the national farmgate agricultural P surplus for NI was 12.3 kg P ha^−1^, up from a low of 8.7 kg P ha^−1^ in 2008 (Fig. 1A). The 2008 surplus was achieved following implementation of the EU Nitrates Directive (91/676/EEC) in 2004 and Phosphorus (Use in Agriculture) Regulation 2006 (Northern Ireland), which helped reduce the P surplus from 17.7 kg P ha^−1^ in 2003, without negative impacts on agricultural productivity, likely owing to the considerable P buffering capabilities of some agricultural soils (Sattari et al., 2012). Within NI, the stated P surplus objective in the government's roadmap for improving farm nutrient efficiency and profitability is 5 kg P ha^−1^ yr^−1^ (DAERA, 2016); however, in recent years the agri-food industry has moved further away from achieving this target. Despite continuing improvements in P use efficiency, the increase in the P surplus since 2008 has largely been due to the gradual increase in the use of imported feed from 13.4 to 16.8 kg P ha^−1^ between 2008 and 2017, and a slight increase in inorganic P fertiliser use from 3.1 to 4.5 kg P ha^−1^ during the same period (DAERA, 2016, 2019).

Mirroring the initial improvements in lowering the national P surplus, there has been a significant reduction in the P concentrations recorded in rivers across NI (Barry and Foy, 2016). However, continued annual monitoring suggests that from 2012 this trend is now reversing (DAERA, 2018a) with river P levels increasing (Fig. 1B). Currently 67% of NI rivers fail to meet EU Water Framework Directive (WFD) targets for ‘good’ or ‘high’ water quality (DAERA, 2018a). Phosphorus inputs from agriculture are the single biggest cause of failure to meet the WFD targets, with 39% of NI waterbodies below the targets required for good water quality due to elevated P levels. The challenges of achieving the WFD targets will likely increase in coming years with increasing demand for food and changing consumption patterns, changes in the policy environment as the UK leaves the European Union (EU) and the ambition for expansion within the NI Agri-Food industry, as detailed within the *Going For Growth 2020* strategy, which includes targets of a 60% increase in turnover, 15% increase in employment, 60% added value on produce and a 75% increase in exports (Agri-Food Strategy Board, 2013).

### 2.2 Substance flow analysis and system boundary

The system boundary for this SFA is the geographical border of Northern Ireland and focuses on the flows and stores of P relevant to the NI food system. The SFA is adapted from a model P SFA system for Austria presented by Egle et al. (2014). It is comprised of ten processes that represent the main stages of the food system in NI and 40 flows that represent the movement of P into and out of the system and between the processes. The SFA presented in this paper is for the year 2017 as this was the most recent year with the most available full data sets. Where data for this year was not available, the nearest year's data was used. Data for the flows of material in the food system were obtained from various reliable sources, starting with published national statistics or industry annual reported data. Where these were not available, either previously published scientific data was used or expert opinion was sought from relevant stakeholders. The P contents of the materials were taken and cross-referenced from various published literature sources and converted to elemental P where necessary. All flows are thus presented as tonnes of elemental P per year.

The imports, exports, flows and losses of all significant materials containing P relevant to the food system were identified. Additional non-food imports of P were included that subsequently became part of a system flow, i.e. P in detergents and P added to water for the purpose of preventing plumbosolvency to meet regulations on the permitted levels of lead in drinking water dissolved from old piping, both of which enter wastewater management. Phosphorus from atmospheric deposition, seeds and wool were omitted from the SFA as they were considered too small a flow of P to be of relevance to this SFA. There are several small anaerobic digestion (AD) plants within NI that are fed predominantly with cattle slurry, manure and grass produced on farm. The digestate produced from these AD plants is spread back onto the farm of origin or locally and thus represents a local, internal cycle that would remain in balance. Therefore, these small on farm flows of AD were omitted from this SFA and only the large-scale commercial AD plants in NI were included.

Detailed descriptions of each process, P contents and data sources used for the associated flows are presented in Supplementary Material section 1.1 and Table S1. The free software STAN was used to undertake the P balance and visualise the P SFA (Cencic and Rechberger, 2008).

### 2.3 Data uncertainty

The inevitable variability in data quality used for any SFA mean appropriate assignment of uncertainty to the data used for flows is important (Laner et al., 2014). This study utilises a systematic approach fully described in Zoboli et al. (2016), which was initially developed by Laner et al. (2016). In brief, an evaluation score is assigned to a range of quality indicators that are translated into co-efficients of variation (CVs) for that quality indicator. The quality indicators described by Zoboli et al. (2016) and used here are: *Reliability, Completeness, Composition, Temporal correlation, Geographical correlation* and *Further correlation*. A final quality indicator, *Expert judgement* is used where expert opinion is used to assign a flow. Each quality indicator is assigned a score between 1 and 4 where 1 represents the most reliable data and 4 the worst, though *Composition, Temporal, Geographical* and *Further correlation* are subject to a further sensitivity assessment based on the subjective opinion of the user. Three sensitivity levels are available from *Highly sensitive, Sensitive* to *Not sensitive,* which then determine the CV assigned to each score. STAN uses the assigned data uncertainty to perform nonlinear data reconciliation based on the method of weighted least squares to find the best fitting values and their uncertainties, which are used to compute an adjusted CV% using methods described fully in Cencic (2016) and Brunner and Rechberger (2016). This means the model output data will most likely differ to the inputted flow data. Inputted and STAN reconciled flow data and CV%’s for this SFA are shown in Table 1. The mean% change for all flows between inputted and reconciled data was −0.14%, giving a high degree of confidence that the data collated to undertake this SFA was suitably accurate. For reasons of clarity, the flow values referred to in text are presented without their uncertainty and values have been rounded to significant figures in relation to their uncertainty (Taylor, 1997).

**Table 1:** Summary of the processes and flows used for the SFA showing the inputted P flow for 2017 and the assigned CV% and the STAN reconciled P flows and adjusted CV%. (WwTW = wastewater treatment works).

Process	Flow number	Flow name	Inputted data		STAN reconciled data		% difference in P flow
			P flow t yr^−1^	CV%	P flow t yr^−1^	CV%	
1. Animal husbandry	F1.01	Import live animals	414	11	414	11	0.04%
	F1.02	Export live animals	325	11	325	11	−0.03%
	F1.03	Animal products	5244	11	5212	11	−0.61%
	F1.04	Manure to fields	21,550	11	20,380	9	−5.43%
	F1.05	Manure to waste	420	15	420	15	−0.11%
	F1.06	Export animal manure	466	15	465	15	−0.08%
	F1.07	Animal slaughter waste	1592	15	1585	13	−0.42%
	F1.08	Animal husbandry to waste	2	11	2	11	0.00%
2. Crops and grass	F2.01	Crop products	1093	11	1093	11	−0.02%
	F2.02	Silage, hay and grazing	15,669	11	15,900	10	1.47%
	F2.03	Agriculture to water bodies	942	25	942	25	0.00%
	F2.04	Crop farming to waste	3	11	3	11	0.00%
3. Food, feed & fertiliser processing	F3.01	Import fertiliser	4370	10	4367	10	−0.08%
	F3.02	Import animal feed	11,768	15	11,713	11	−0.47%
	F3.03	Import food	1066	25	1065	25	−0.12%
	F3.04	Fish landings	349	10	348	10	−0.01%
	F3.05	Fertiliser to crops	4370	10	4373	10	0.08%
	F3.06	Export animal feed	790	11	790	11	0.02%
	F3.07	Export food	4388	25	4409	23	0.49%
	F3.08	Animal feed to livestock	11,911	11	12,075	9	1.38%
	F3.09	Food to consumption	1308	6	1289	8	−1.45%
	F3.10	Food processing to waste	529	11	529	11	−0.01%
	F3.11	Food processing to crops	9	11	9	11	0.00%
	F3.12	Industry to WwTW	298	11	297	11	−0.40%
	F3.13	Industry to water	40	40	40	40	0.01%
	F3.14	Import non-food	497	25	497	25	−0.06%
	F3.15	Non-food to consumption	497	25	483	21	−2.85%
4. Consumption	F4.01	Consumption recycled waste	278	11	279	10	0.25%
	F4.02	Consumer landfill waste	107	11	107	11	0.12%
	F4.03	Consumption to WwTW	1142	15	1137	8	−0.49%
	F4.04	Consumption to septic tanks	248	15	249	11	0.28%
5. Wastewater management	F5.01	Biosolids to waste management	998	11	1011	9	1.31%
	F5.02	Biosolids to crop farming	60	11	60	11	0.08%
	F5.03	WW to water	359	15	362	14	0.91%
6. Waste Management and Bioenergy	F6.01	Waste management to landfill	1	11	1	11	0.00%
	F6.02	Waste management to crops	348	11	348	11	0.02%
	F6.03	Waste management export	2133	20	2140	11	0.34%
	F6.04	Waste to domestic market	258	20	258	20	0.04%
	F6.05	Biosolid ash to landfill	1081	11	1082	11	0.05%
7. Landfill	Sink		n/a	n/a	n/a	n/a	n/a
8. Water bodies	Sink		n/a	n/a	n/a	n/a	n/a
9. Septic tanks	F9.01	Septic tanks to water	184	15	184	15	0.00%
10. Domestic Market	Sink		n/a	n/a	n/a	n/a	n/a

## 3 Results and discussion

### 3.1 National P balance

**Fig. 2:**
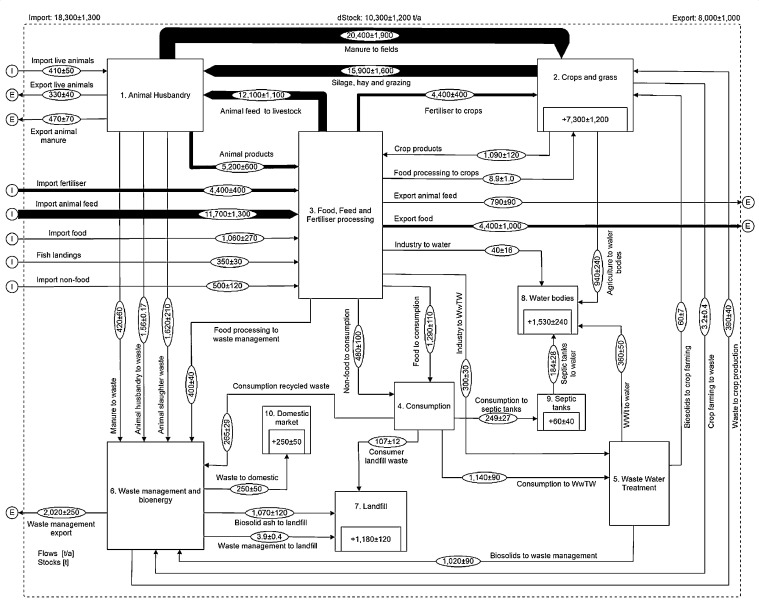
Phosphorus flows in the Northern Ireland food system for 2017, all values have been reconciled by STAN and are shown as tonnes P per year ± standard error. Values shown within a process represent the annual accumulation (sink) within that process and values have been rounded to significant figures in relation to their uncertainty. A detailed description of the processes and flows can be found in the supplementary information file.

Like most countries globally, NI imports P to satisfy the P demand for its food production systems. The SFA shown in Fig. 2 reveals that in 2017 NI imported a total of 18,300 t (9.8 kg cap^−1^) of P as animal feed (64%), mineral fertiliser (24%), food (6%), fish landings (2%), live animals (2%) and non-food P (3%). Exports of P were 8000 t P (4.3 kg cap^−1^ yr^−1^) primarily as food products (55%), the rest through waste management (25%), animal feed (10%), manure (6%) and live animals (4%), leaving a system P surplus of 10,300 t which equates to a net national consumption of 5.5 kg P cap^−1^. This is much greater than the average UK net P consumption of 1.9 kg cap^−1^ reported by Cooper and Carliell-Marquet (2013) for 2009, however this was an exceptional year, with mineral P fertiliser inputs substantially reduced across the UK owing to large price increases. An average net national P consumption of 4.7 kg cap^−1^ reported for the EU 15 (Ott and Rechberger, 2012) and 4.9 kg cap^−1^ reported for the EU 27 in 2005 (van Dijk et al., 2016) would suggest that NI net P imports are slightly above average.

The food system efficiency can be estimated from the sum of the P in food consumed and ‘commodities’ exported divided by the P imports used to produce those goods. The NI food system produced 5700 t of P in food for home consumption and export, 790 t in exported animal feed and 330 t in exported live animals from a total of 17,800 t of P imports (11,700 t feed, 4400 t mineral fertiliser, 1060 t food, 410 t live animals and 350 t in fish landings) giving a food system P efficiency of 38%. Reported food system P efficiencies in the rest of the EU in 2005 ranged from 22 to 139% with a whole EU 27 average of 38% (Withers et al., 2020), whilst a value of 46% was reported for the UK in 2009 (Cooper and Carliell-Marquet, 2013). The low food system P efficiency in NI reflects the high prevalence of livestock agriculture, which is inherently less efficient in converting P to food than crop based agriculture (Metson et al., 2012).

### 3.2 Agriculture

#### 3.2.1 Livestock

Livestock in NI consumed a total of 28,000 t of P in 2017, 15,900 t (57% of total) as grass either grazed or as silage/hay and 12,100 t (43%) as animal feed. Total P output in food produce from the livestock sector as meat (dressed carcass weight [dcw]), milk and eggs was 5200 t giving a P efficiency for the livestock sector of 19%. This is similar to the livestock efficiencies reported for the whole of the UK of 17% for 2009 (Cooper and Carliell-Marquet, 2013) or 22% for 2005 (data calculated from van Dijk et al. (2016)), but is considerably lower than other livestock intensive countries such as the Netherlands, 52% and Denmark, 36%, (both calculated from van Dijk et al. (2016)). The livestock sector efficiency in NI is perhaps limited by the dominance of ruminant-based production, which requires more land base for a given output. A more specific breakdown by livestock type (Table 2) shows that the biggest flow of P in the livestock sector in NI is in the grass and silage (14,000, 88% of total) and supplementary feed (5900 t, 49%) consumed by cattle. Their produce contained 60% of the total livestock P output (meat 950 t and milk 2170 t) giving a P efficiency of 16%. As a consequence of the high P intake, cattle produced 81% of the P contained in all livestock manure and slurry. The most P efficient sector was pigs with 560 t (11% of total) of P in its meat produced from 1330 t (11%) feed P intake giving a P efficiency of 42%. Pig manure contained 1090 t of P, around 5% of total manure P production. Poultry produce (meat and eggs) contained 1480 t of P (28% of total) from 4240 t (35%) of feed P giving a P efficiency of 35%. Poultry manure contained 1840 t (8%) of P, though nearly half of that was exported from NI either directly or through waste management, meaning poultry accounted for 5% of manure P inputs to the soil in NI. The sheep sector was the least P efficient with 110 t (2% of total) of P in meat produce from 2110 t of grazing (11%) and feed (3%) P input giving a P efficiency of 5%. Sheep manure contained 1870 t of P, around 9% of total manure P produced.

**Table 2:** Details of phosphorus flows and sector efficiency for different livestock types, all flow values are tonnes P per year for 2017.

Livestock	Feed P	Silage/ grazing P	Manure P to soil	Manure P exported	Manure P to waste management	Meat P	Milk P	Egg P	% P efficiency
Cattle	5900	14,000	16,500	n/a	n/a	950	2170	n/a	16
Pig	1330	n/a	1090	n/a	n/a	560	n/a	n/a	42
Poultry	4240	n/a	950	470	420	1310	n/a	170	35
Sheep	360	1750	1870	n/a	n/a	110	n/a	n/a	5
Other	240	160	44	n/a	n/a	n/a	n/a	n/a	n/a
Sum	12,070	15,910	20,454	470	420	2820	2170	170	

#### 3.2.2 Soil

The biggest flow of P in the soil based agricultural system for 2017 was the 20,400 t of P in all livestock manure that was either deposited directly during grazing or spread on agricultural land. This represented 81% of all P inputs to soil, with an additional 4400 t (17%) from mineral fertilisers and 460 t (2%) from waste recycling products. Total P inputs to agricultural soil were therefore 25,200 t or 24.7 kg ha^−1^ when averaged over the entire agricultural land in NI or 29.6 kg ha^−1^ excluding rough grazing which is unlikely to receive any P input other that small amounts from direct manure deposition from livestock. DEFRA's national P balance for the UK in 2017, which also excludes rough grazing, gives a national average P input of 24.3 kg ha^−1^ (DEFRA, 2018), meaning NI P inputs to the soil are over 5 kg ha^−1^ greater than the UK average. These high P input levels are perhaps reflective of historical challenges around the use of cattle slurry, where application rates are often driven by storage capacity and land area availability rather than actual agronomic need (Smith et al., 2001). Total P output from soil based agriculture in both crops and grass was 17,000 t (16.7 kg ha^−1^) giving a cropland P efficiency of 67%, which is consistent with an average P efficiency of 68% estimated for grass production in the NI dairy sector between 2009 and 2014 (Adenuga et al., 2018). The remaining P not taken up by grass and crops either accumulated in the soil (7300 t) or was lost to water (940 t). The surplus P accumulation in the soil equates to 8.5 kg ha^−1^ excluding rough grazing. van Dijk et al. (2016) reported a soil P surplus of 4.2 kg ha^−1^ for the UK, and 4.9 kg ha^−1^ for the EU 27, in 2005. Cooper and Carliell-Marquet (2013) reported an average P surplus of 3.5 kg ha^−1^ for arable and grassland in the UK or 4.3 kg ha^−1^ just for grassland in 2009, though the low mineral fertiliser P input for that year may mean the surplus is lower than typical. DEFRA report a P surplus of 6.2 kg ha^−1^ for the whole of the UK in 2017, however, this will include a higher proportion of arable farming, where P offtake would be expected to be higher than grassland (AHDB, 2017); therefore the P surplus for NI appears to be particularly high. Excess applications of P above crop requirement is one of the key drivers of impaired water quality (Withers et al., 2014) and NI appears to be no exception to this. The P losses to water from agriculture in NI reported here represent 62% of total national P flows to fresh and coastal waters.

### 3.3 Food processing and consumption

The food processing sector received a total of 6800 t of P, 5200 t (77%) from livestock sector food products, 1060 t (16%) from imported food, 350 t (5%) from fish landings and 190 t (3%) from crops grown in NI for human consumption. The food produced from this contained 5700 t of P giving a sector P efficiency of 84% which is consistent with the EU 27 average of 80% reported by van Dijk et al. (2016). Of the total P contained in food products, 4400 t (77%) of food produce P was exported and 1290 t (23%) entered domestic markets, which, when the 480 t of non-food P ‘consumed’ as detergent and plumbosolvency P is added, gives a per capita P consumption of 0.94 kg yr^−1^. When food wasted from households and the food service sector and non-food P are excluded, the actual per capita food P intake for NI was 0.48 kg cap^−1^ yr^−1^ which is similar to the UK national average of 0.47 kg cap^−1^ yr^−1^ (Gregory, 1990) and the 0.44 kg cap^−1^ yr^−1^ reported for NI by Foy et al. (1995).

### 3.4 Waste and wastewater management

In 2017 waste water treatment works (WwTW's) in NI received 1440 t of P in wastewater (Table 3), 730 t (51%) from food consumed by the population of NI, 240 t (16%) from detergent P, 170 t (12%) in plumbosolvency P added to the drinking water consumed by the population and trade effluent P contributed 300 t (21%). Around 75% (1070 t) of P was removed in the biosolids during wastewater treatment, 1020 t of which was sent to waste management for incineration and 60 t was recycled back to agricultural land. 360 t of P was lost from WwTW's into fresh and coastal waters of NI, representing 24% of total food system losses to water. Septic tanks received a total of 249 t of P (Table 3), 160 t (64%) from food consumption, 51 t (21%) detergent P and 37 t (15%) of plumbosolvency P. 184 t of septic tank P was lost to water giving a discharge co-efficient (the percentage of received P that is lost) of 74% representing 12% of total P losses to water in NI, 65 t of P accumulated within the septic tanks and their soakaways. When WwTW's and septic tanks are combined, the P removal efficiency for all wastewater treatment in NI equates to 66% which is almost identical to the UK national average of 68% reported by Naden et al. (2016).

**Table 3:** Details of phosphorus flows and removal efficiencies under different wastewater treatment technologies in Northern Ireland for 2017. ^a^reported by Naden et al. (2016), ^b^calculated from export co-efficient (Barry and McRoberts, 2016).

Waste water treatment technology	Total P received at each treatment level (tonnes yr^−1^)	% of total received at each treatment	Fraction of P discharged	Total P discharged (tonnes yr^−1^)	% of total discharge
Primary	22	1	0.78^a^	17	3
Secondary	407	24	0.42^a^	171	32
Tertiary	46	3	0.35^a^	16	3
Advanced	964	57	0.16^a^	154	28
Septic tank	248	15	0.74^b^	184	34
Sum	1687			542	

Primary, secondary, tertiary and advanced wastewater treatment technologies represent increasing levels of P removal to meet varying regulatory standards, see Naden et al. (2016) and references within for more detail.

The waste management sector received a total of 3740 t of P in 2017. The largest portion of this was 1620 t of P in animal slaughter waste, 90% of which was exported outside NI for processing, the remaining 10% entering the pet food market in NI. Food waste collected from food processing, households and the food service sector contained 670 t of P, approximately 65% of which was processed via AD and 35% via composting, around 30% and 20% of which was exported respectively. A further 40% of the compost entered domestic markets meaning only 390 t of food waste P was recycled to agriculture in NI. A significant volume of the poultry manure produced in NI is treated via AD, receiving 420 t of P nearly all of which was exported after further processing into horticultural compost products. As well as the 1020 t of P in incinerated biosolids, landfill also directly received 107 t of P from food waste in domestic municipal waste. Therefore, 54% of total received P in waste was exported outside NI, 32% was landfilled, 10% was recycled to agriculture and 7% entered domestic markets highlighting the current lack of P circularity in the NI food system.

### 3.5 Opportunities for improved P sustainability

This SFA has provided key information on the current flows, accumulations and losses of P within the different sectors of the NI food system that is essential for prioritizing the direction of change required to improve the region's P sustainability. Similar to the Republic of Ireland (RoI), the NI economy is heavily reliant on a thriving and profitable agriculture centred around ruminant livestock farming on productive pasture in a mild, wet climate. High P inputs relative to productive output are typical of regions with high animal densities (Withers et al., 2020), and the predominance of ruminant livestock have led to a regional food system with a very low P efficiency (38%). As highlighted in Table 2, P efficiency in the ruminant sector is lower than in the non-ruminant sector, and this has been reflected in other SFA studies; for example (based on 2005 data of van Dijk et al. (2016)), system P efficiency in the RoI was only 22% compared to Belgium (59%), The Netherlands (66%) and Denmark (44%) with a much larger non-ruminant livestock industry.

The low P efficiency in NI leads to a large amount of unused P that either accumulates in the soil as a P surplus (7300 t yr^−1^), is lost to fresh and coastal waters (1530 t yr^−1^), accumulates in landfill (1180 t yr^−1^), or is exported outside the region as waste (2020 t yr^−1^). Surplus P accumulation in NI soils has been ongoing since at least 1925 when the national balance for agriculture was estimated to be 3.5 kg P ha^−1^, rising to a high of 24 kg P ha^−1^ in 1962 (Foy et al., 2002) before gradually declining to a surplus of 8.7 kg P ha^−1^ recorded in 2008 (DAERA, 2016). This accumulated ‘legacy’ soil P from past P inputs is reflected in the high percentage of soils with excess P in NI (Higgins et al., 2020), and has been a long-term and increasingly significant source of P loss in land runoff (Cassidy et al., 2019; Foy et al., 2003, 1995). Together with P discharges from WwTW, diffuse P inputs to waterbodies, which now represent 62% of all P losses to water, are the major cause of poor water quality and ecological status in NI. For example, over 70% of NI lakes are classed as eutrophic, and in the largest lake Lough Neagh, an estimated >200 tonnes of accumulated P is released into the water column from lake sediment annually (Gibson et al., 2001).

Options to improve P sustainability in NI must therefore address the low P efficiency and the continuing system P surplus that poses such a threat to NI water resources and ecosystem biodiversity. Suitable targets for improving the sustainability of P use in a food system include increasing P circularity to avoid importing fresh P, minimising P loss and targeting a zero P balance by matching food system P demand with supply.

#### 3.5.1 Increasing P circularity

Livestock manure is the largest P flow in the NI food system, but most of this is already recycled back to agricultural soil and is the major contributor to the soil P surplus. Opportunities for increased P circularity therefore rest with the waste management sector. On mass balance, if the 3740 t of P currently received by the waste management and bioenergy sector was effectively recycled, it could meet 22% of the crop and grass P demand for NI, directly replacing 88% of mineral fertiliser imports. The relative agronomic efficiency (RAE) of recycled P products compared to mineral P fertilisers is important when considering fertiliser replacement potential (Hamilton et al., 2017). However, novel waste processing technologies, for example animal slaughter waste (Darch et al., 2019) and struvite from human waste (Talboys et al., 2016), can equal mineral P fertiliser performance suggesting potential full fertiliser replacement from different waste streams is feasible (Huygens and Saveyn, 2018). In practice, public perception concerns ranging from odour when stored or spread, to regulatory, licensing and food safety requirements can compromise acceptability, limiting the land area available to spread and will often inflate the cost and management requirements when using such materials as fertilisers. For example, the majority of wastewater sludge biosolids in NI are incinerated rather than spread because of the regulatory restrictions on their application to pasture, and land application of aerobic and anaerobic sludges from agri-food processing waste treatment must be licensed under regional legislation (Waste Management Licensing Regulations (Northern Ireland), 2003), which increases the management cost. However, the more fundamental problem for the regions P sustainability is that recovery and further recycling of P from the waste management sector will only add to the animal manure P burden already circulating within food system (Withers et al., 2018). In NI, this animal manure P burden already exceeds system P demand (Fig. 2), and will therefore only exacerbate the NI P surplus.

#### 3.5.2 Reducing P losses

Current P discharge from wastewater management in NI (WwTW's and septic tanks) is around 34% of received P (Table 3), and although over half of received P is treated by advanced P stripping technology, around two thirds of P lost in wastewater treatment comes from facilities with poor removal efficiency, particularly those with secondary treatment and septic tanks typical of small and rural communities. In common with other rural populations (e.g. Yates et al., 2019), opportunities for using advanced P removal technologies, such as struvite recovery, in wastewater treatment are not yet cost effective, though different nature based technologies such as constructed wetlands (Vymazal, 2011) or short rotation coppice willow plantations grown for bio-resources (McCracken and Johnston, 2015) could provide cost effective opportunities for reducing P loss to water in these situations.

Losses of P to water from agricultural land in NI include those from freshly applied manures and mineral fertilisers, and soil accumulated P from historic over-application (Cassidy et al., 2017; Doody et al., 2012). There is some evidence that measures that limit manure and fertiliser use by targeting the farm-gate P balance have reduced losses of P to waterbodies in NI (Barry and Foy, 2016); however in NI river P concentrations are starting to increase again (Fig. 1), and in many other regions the outcomes from voluntary and regulated approaches to best practice management of P inputs continue to remain uncertain, as P targets required for eutrophication control in rivers and lakes are not being achieved (Kleinman et al., 2015).

#### 3.5.3 Targeting a zero P balance

Achieving a zero system P surplus by matching P supply to P demand would require the most change in NI agriculture. Since the P supplied by animal manures alone already exceeds total crop P demand by nearly 20%, inputs of manure P would need to be reduced by around 3390 t P yr^−1^, or by 8220 t P yr^−1^ (48%) if current mineral fertiliser and recycled P usage rates remained the same, to match current system P offtake. In practice, balanced P applications to agricultural land using just livestock manure are constrained by the available land area; a large proportion (ca. 68%) of the NI agricultural area is classed as a ‘Less Favourable Area’ with agricultural activity constrained by high soil moisture, frequent rainfall and slope, and many lowland catchments have significant proportions of critical source areas of runoff P loss where repeated manure applications would not be advised (Cassidy et al., 2019). Reducing manure P production could be addressed by cutting livestock numbers and associated reductions in total feed imports. Livestock density is the main driver for food system imports across Europe (Withers et al., 2020) and reducing livestock numbers to balance P inputs and outputs from the available land could be a key strategy for improving agricultural P efficiency globally (Schroder et al., 2011). However, with the case of NI, a manure P reduction of 20% is approximately equivalent to the entire manure P output from the sheep, pigs and poultry population or around a quarter of the cattle population. With the NI agri-food industries ambition to expand these sectors (Agri-Food Strategy Board, 2013), and given how significant livestock based food production currently is to agriculture in NI, large reductions in livestock numbers are unlikely to occur except through regulation.

Reducing the P content of the manure produced is another option which would also help to reduce manure P release to land runoff; for example O'Rourke et al. (2010) observed that a 43% reduction in the P content of the diet of dairy cows resulted in a 61% reduction in manure water soluble P and consequently a 58, 74, and 48% reduction in dissolved reactive P in overland flow, in summer, winter and spring, respectively. The average supplementary animal feed P content (excluding grass and silage) calculated in this SFA was 0.46%. To achieve a 20% reduction in manure P content, taking account of the fact that over half of manure P comes from grass and silage consumption with an assumed P concentration of 0.33%, average supplementary animal feed P concentrations would have to drop to 0.25%. Given that minimum adequate dietary P levels in dairy cattle are around 0.35 to 0.42% (Ferris et al., 2010), and opportunities to further reduce feed P in pigs and poultry beyond current phytase supplementation without welfare issues are limited (Liu et al., 2019), such reductions in feed P content would not be sustainable. In 2005, the Northern Ireland Grain Trade Association entered into a voluntary agreement with the government to reduce the P content of animal feeds by 10% (DAERA, 2019). However, since this agreement there has been a significant increase in the quantity of supplementary concentrates fed to livestock, with inputs increasing from 15 kg P ha^−1^ in 2005 to 16.8 kg P ha^−1^ in 2017 (DAERA, 2019). Consequently, the agri-food industry in their recent Sustainable Agricultural Land Use Management Strategy (Expert Working Group on Sustainable Land Management, 2016), is now advocating to nearly double the average grass dry matter intake (from 5 to 9.5 t ha^−1^) in the grass-based livestock sector, which will help to reduce the intake of supplementary feed.

Exporting slurries and manures outside of the NI food system could resolve the P surplus problem, especially as the P demand in arable areas of the UK is high (Bateman et al., 2011). However, difficulties in moving these materials any significant distance currently make this uneconomic and impractical, especially as cattle are by far the largest producers of watery manures and slurries in NI. Some (ca. 25%) of the poultry manure produced in NI is exported directly, reflecting its relatively low moisture content and high nutrient value compared to other livestock manures. A further quarter of NI poultry manure is processed via a unique AD technology, the dry fraction of which captures 95% of the P, can be easily transported and is currently exported outside NI in horticultural products after further processing (Anon, Stream BioEnergy pers. comm. Sept 2019). Though this currently only represents around 2% of total manure P production, it demonstrates the potential for new processing technologies to manage manure P. Some cattle slurry and manure is processed on farm via AD in NI for electricity generation via biogas production, though the products of this current technology are similar to slurry and are still bulky and difficult to transport, and are returned to the soil locally. New processing technologies that allow physical and chemical separation of the nutritional components of slurry and AD products (e.g. dewatering, filtration, thermochemical conversion) could make future transport and export of manures more viable (e.g. Porterfield et al., 2020). They could also provide “waste to wealth opportunities” and transform perceptions so that P containing waste streams can be seen as valued resources which can be transformed into circular and sustainable products.

## 4 Conclusion

This SFA clearly demonstrates the significant influence of the livestock sector on P flows in the NI food system. Animal feed P is by far the largest import into the system and the resulting livestock manure P the largest internal flow within the system and the largest contributor to the national P surplus. Overall food system P efficiency is consequently low and the eutrophication threat from continued surplus P accumulation in soils and waterbodies is high. Manure P loading to soil alone already exceeds crop P demand by 20%, and while there are some additional opportunities to recover P from the waste management sector to improve P circularity, this would only add to the P burden circulating within the system, increase the P surplus and the threat of P loss in land runoff. Diffuse P loss from agriculture is now the major threat to water security in NI representing 62% of P losses to water bodies. Balancing the P inefficiencies inherent to a livestock dominated food system by limiting feed P imports and livestock density with the economic reality of a productive agricultural sector is a key challenge for any livestock dominated food system. Priorities for the NI food system should therefore target reducing P imports by limiting mineral P fertiliser use, reducing livestock P demand where possible and/or management of surplus P by exporting of appropriately processed bioresources. This SFA will provide the basis for future discussions with relevant stakeholders to improve P stewardship in the NI food system.

## CRediT authorship contribution statement

**S.A. Rothwell:** Investigation, Methodology. **D.G. Doody:** Investigation, Writing - review & editing, Funding acquisition. **C. Johnston:** Investigation, Writing - review & editing. **K.J. Forber:** Investigation. **O. Cencic:** Software, Writing - review & editing. **H. Rechberger:** Writing - review & editing. **P.J.A. Withers:** Writing - review & editing, Funding acquisition, Supervision.

## Declaration of Competing Interest

The authors declare that they have no known competing financial interests or personal relationships that could have appeared to influence the work reported in this paper.

## Appendix Supplementary materials

Image, application 1
