# Supplementary material for: Phosphorus stocks and flows in an intensive livestock dominated food system
Source: Resour Conserv Recycl. 2020 Dec;163:105065. doi: 10.1016/j.resconrec.2020.105065 (PMC7534034; doi:10.1016/j.resconrec.2020.105065)
Supplement: Supplementary file 1 [file mmc1.pdf]

Phosphorus Stocks and Flows in an Intensive Livestock Dominated Food System

Shane A Rothwell[a], Donnacha G Doody[b], Chris Johnston[b], Kirsty J Forber[a], Cencic O[c], Rechberger H[c], Paul J A Withers[a]

[a] Lancaster Environment Centre, Lancaster University, Lancaster, UK

[b] Agri Food and Biosciences Institute, Belfast, Northern Ireland

[c] Institute for Water Quality and Resource Management, TU Wien, Vienna, Austria

Supplementary material

1.1 Details of processes and flows

Process 1 is *Animal Husbandry* which includes all livestock grown for the purpose of food production or breeding, notably, beef and dairy cattle, sheep and lambs, pigs and poultry. Eight flows are associated with this process including the P imported and exported in live animals (F1.01 and F1.02 respectively). Livestock numbers for 2017 were obtained from the Northern Ireland Food Animal Information System (NIFAIS) (DAERA, Pers. Comm. 2019), estimated live weights per animal were calculated from average carcass weights of 343 kg for cattle (AHDB, 2018), 19 kg for lamb (DEFRA, 2018) and 83 kg for pigs (AHDB, 2017a) and respective killing out percentages of 53% , 50% and 75%, these, and the P content of live animals, were taken from Foy et al. (2002). F1.03 represents the flow of P in all animal food products generated in the NI livestock system. 2017 production figures for beef, lamb, pig and poultry meat (as dressed carcass weight (dcw)), milk and eggs were taken from NI 'main product output' statistics (DAERA, 2018c) and P contents of the meat products from Foy et al. (2002) and milk and eggs from McCance and Widdowson (2015). Phosphorus in manure and slurry that ends up in the soil system is shown in F1.04, this includes excreta that is both directly deposited by grazing animals and that which is spread on agricultural fields from housed animals. Annual P excretion co-efficients for different livestock classes (DEFRA, 2006; Russell et al., 2008), were multiplied by the livestock population from the 2017 annual livestock census (DAERA, 2018b).

Phosphorus in poultry manure that enters the waste management and bioenergy sector as commercial AD feedstock is shown in F1.05 and P in poultry manure exported outside of NI for land spreading or bioenergy is shown together in F1.06. Volumes and P content of these flows were estimated by expert opinion of professionals in the NI bioenergy and poultry manure management sector. Phosphorus in by-products of the animal slaughter process that do not enter the human food chain is represented by F1.07. The amount of slaughter waste was calculated from dcw production figures and killing out percentages for cattle, lamb and pigs described above plus a killing out % of 0.7 for poultry and P content from Foy et al. (2002). Phosphorus in other animal husbandry waste (F1.08), typically fallen stock, was calculated from NI 2017 'waste summary returns' (NIEA, Pers. Comm. 2019) and an average livestock P content from Foy et al. (2002).

Process 2 is *Crops and Grass* that represents the soil-based agriculture that produces all tillage crops, horticultural produce and grass for both grazing and silage/hay. Four flows are associated with this process, including the P in all food and feed crop products from tillage agriculture and horticulture (F2.01). Quantities of crop products were from NI 2017 'main product output' statistics (DAERA, 2018c) plus an additional 50% production for wheat and barley to account for grain that is produced and fed locally that does not appear in national statistics (NIGTA, Pers. Comm. 2019). The P content of the crop products came from AHDB Nutrient Management Guide RB209 (AHDB, 2017b) or the AFBI nutrient balance for 2017 (AFBI Pers. Comm 2019). The P in all grass-based production (F2.02), included grass harvested for animal fodder (silage and hay) and direct grazing. 2017 production figures for silage and hay came from national statistics (DAERA, 2018c) and the P content from RB209 (AHDB, 2017b). Grazing P was estimated from the P nutritional requirements (DEFRA, 2006) of the population of ruminant animals and horses (DAERA, 2018b) minus the P supplied from silage and hay and supplementary animal feed. Diffuse losses of P from agricultural land to waterbodies (F2.03) were calculated from annual P export co-efficients reported by Smith et al. (2005) for NI of 4.88 kg/ha (crops), 0.8 kg/ha (grass) and 0.48 (rough grazing) multiplied by corresponding 2017 crop areas (DAERA, 2018a). The export co-efficients for grass and rough grazing were estimated from the

area weighted average of values for ‘good pasture’, ‘poor pasture’ and ‘mixed pasture’ (grass) and ‘agricultural natural vegetation’, ‘natural grassland’ and ‘moor and heath’ (rough grazing) reported by Smith et al. (2005), respectively. Phosphorus in waste from crop and grass farming going to waste management is represented in F2.04, amounts of waste were taken from NI 2017 ‘waste summary returns’ (DAERA, Pers. Comm. 2019) and the average P content from crop products. The process *Crops and Grass* also includes a stock value that represents the annual accumulation or surplus of P in that process, i.e. the excess P applied above the requirements of the crops and grass grown that accumulates annually in the soil.

Process 3 is *Food, Feed and Fertiliser Processing* that deals with the imports, exports and internal movements of food, feed and fertiliser P, including the processing of food and feed products and any associated waste flows. There are 15 flows associated with this process. The import of mineral P fertilisers and its subsequent flow to *Crops and Grass* (F3.01 and F3.05 respectively) was calculated from national fertiliser delivery statistics for 2017 (DAERA, 2019b), both import and fertiliser use were assumed to be the same and used within the year of delivery. The P contained in imports and exports of animal feed and the flow of feed to *Animal Husbandry* are shown in flows F3.02, F3.06 and F3.08 respectively. The P fed in livestock feed was calculated from the quantities of raw ingredients used in 2017 by the animal feed sector (DAERA, 2019a) plus the additional 50% of local wheat and barley production fed to livestock (NIGTA, Pers. Comm. 2019). The P content of the feed ingredients came from Feedtables.com (INRA, 2019b) or feedipedia.org (INRA, 2019a) and supplementary P added to pig and poultry diets as monodicalcium phosphate (MDCP) was calculated from expert opinion on industry dietary inclusion rates (AFBI, Pers. Comm. 2019). Imports of animal feed P were calculated by subtracting the animal feed ingredient usage that is met by local production (NIGTA, Pers. Comm. 2019) from the total used, the remaining feed ingredients used were assumed to be imported. Phosphorus in the imports and exports of food is represented by flows F3.03 and F3.07 respectively. Food P imports were calculated from HMRC 2017 food import figures (HMRC, 2019) and an average food P content calculated from McCance and Widdowson

(2015). Food exports were estimated from DAERA statistics (DAERA, 2018d) that report that 76% of NI food production in 2017 was exported, thus 76% of the total P from F1.03 animal products, F2.01 crop products and F3.04 fish landings were assumed to be representative of food P exports. P entering the NI food system from fish landings was calculated from recorded weights of catch in 2017 (DAERA, 2019c) and a P content for each fish type from McCance and Widdowson (2015). Phosphorus in food moving to households and the food service sector (F3.9) was calculated from estimated daily per person intake of 1352 mg P in food (Forber et al., 2020) multiplied by the population of NI in 2017 (NISRA, 2018), plus the P in flows of waste food (F4.01 and F4.02) described below. F3.10 represents the P in waste materials from food processing which typically go to *Waste Management and Bioenergy* as feedstock for AD and composting. Amounts and destination of food processing waste came from NIEA 2017 'waste summary returns' (DAERA, Pers. Comm. 2019) and the average food P content from McCance and Widdowson (2015) and Davis and Rudd (1999). Volumes and P content of food processing wastes that are spread directly to agricultural fields (F3.11), as activated sludge from the dairy and meat-processing sector, came from NIEA 2017 'waste summary returns' (DAERA, Pers. Comm. 2019) and Davis and Rudd (1999) respectively. Industrial flows of P either to WwTW's or directly discharged under consent are shown in flows F3.12 and F3.13 respectively. Industrial P to WwTW's was calculated from reported 2017 trade effluent volumes (NIW, 2017) and the P content of trade effluent from Puchlik and Struk-Sokolowska (2017). An estimate of direct industrial P discharge to water was taken from Smith et al. (2005). The import of non-food P and subsequent flow to *Consumption* is shown in flows F3.14 and 3.15 respectively; this represents the P contained in both detergents and that added to drinking water under legislation to prevent plumbosolvency. Detergent P was calculated from per capita annual P load of 0.154 kg cap^{-1} yr^{-1} (Richards et al., 2015) multiplied by the population 2017 of NI (NISRA, 2018) and plumbosolvency P was calculated from reported treatment volumes at a rate of 1 mg l^{-1} (NIW, 2017). It was assumed that the import of non-food P and the flow to *Consumption* were the same.

Process 4 is *Consumption* that includes all food consumed or wasted in households, institutions and the food service sector and is represented by four different flows. Phosphorus in food waste collected at the kerbside for recycling via AD or composting (F4.01) was calculated from NI 2017 'waste summary returns' (NIEA, Pers. Comm. 2019) and an average food P content from McCance and Widdowson (2015). Phosphorus in household food entering municipal waste for disposal at *Landfill* is shown in F4.02 and was estimated from a 2017 survey of household waste in NI (Nesbitt and Devine, 2018) and the same food P content from McCance and Widdowson (2015). Post consumption waste P flows to WwTW's (F4.03) were calculated from the estimated daily food intake P of 1352 mg (Forber et al., 2020) , assuming human P intake equals P excretion, plus non-food P (F3.15) adjusted for the 82% of the population that are connected to the wastewater treatment system in NI (NIW, 2017). The difference between the total population of NI and the population served by the wastewater network was used to estimate the population connected to septic tanks, the same concentration of food and non-food P as F4.03 was then used to calculate the P flow to septic tanks (F4.04).

Process 5 is *Wastewater Management* that represents the processing of wastewater arising from households and industry served by WwTW's and is associated with three flows. F5.01 is the flow of P in biosolids removed at the WwTW's to *Waste Management*. This was estimated from the total P load to WwTW's from food P (F3.9), non-food P (F3.15) and trade effluent P (F3.12) minus the P removed at the WwTW's (see below) and the P in biosolids spread on agricultural land (F5.02). P removal efficiencies for different classes of WwTW's were estimated from published P discharge co-efficients of 0.78 (primary), 0.42 (secondary), 0.35 (tertiary) and 0.16 (advanced) (Naden et al., 2016). The volume of wastewater treated at each class of WwTW was taken from NIW 2017 annual reporting (NIW, 2017), the total calculated P removed at WwTW's being proportional to the removal efficiencies and volumes treated. F5.02 is the flow of P in biosolids spread on agricultural land and was calculated from reported 2017 biosolid usage volumes (NIW, 2017) and an average P concentration for biosolids used for agricultural application (AHDB, 2017b). The P discharged to

waterbodies from WwTW's is shown in F5.03 and was estimated from the discharge co-efficients (Naden et al., 2016) and wastewater volumes used above.

Process 6 is *Waste Management and Bioenergy* that represents the management and processing of organic wastes from all sectors of the food system. Five flows are associated with this process including F6.01 which represents the P contained in the flow of materials to *Landfill* arising from 2017 reported domestic and commercial kitchen waste collection (DAERA, Pers. Comm. 2019) and was assumed to be food derived waste so the same P content from F4.01 was used. The P in both AD and compost that is recycled back to agricultural fields in NI is shown in F6.02, the volume of materials was estimated by expert opinion and the P content derived for F3.10, food processing waste, was used. Total waste management P export, comprised of AD, compost and animal slaughter waste, is shown in F6.03, the volume of materials was estimated by expert opinion and the P content as used previously in F3.10 and F1.07.

Waste management by-products that enter the domestic market as compost or pet food are shown in F6.04, the flow of compost was estimated by expert opinion and the volume of pet food derived from animal slaughter waste also by expert opinion and the same P content used for animal slaughter waste (F1.07). F6.05 represents the flow of biosolid ash to landfill and was derived from F5.01 (P in biosolids arriving at waste management) assuming 100% of biosolid ash is landfilled (NIW, 2017) and no P is lost during the incineration process.

Processes 7 *Landfill*, 8 *Waterbodies* and 10 *Domestic Markets* represent sinks where P is being accumulated or lost from the food system. *Landfill* is a sink representing the annual accumulation of P from waste materials entering landfill. *Waterbodies* represents the annual losses of P to fresh and coastal waters in NI from agriculture, wastewater treatment and industry. *Domestic Markets* is a sink that includes the P contained in pet foods and compost used in domestic gardens. All sink values were calculated by the STAN software and are show in the corresponding process in Figure 2 of the main manuscript.

Process 9 is *Septic Tanks* which includes a STAN calculated sink that represents the annual accumulation of P arising from households in septic tanks. The associated loss of P from septic tanks to waterbodies is shown in F9.01 and was estimated from F4.04 using a P export of co-efficient of 0.55 kg cap^{-1} yr^{-1} reported for 2016 in NI (Barry and McRoberts, 2016).

Table S1: P content and livestock P excretion coefficients used for the NI P SFA

**Relevant flow**	**Item**	**Note**	**P content kg t^{-1}**	**P excretion co-efficient kg head yr^{-1}**	**Reference**
F1.01 F1.02 F1.03	Live animals or livestock meat products as dressed carcass weight	Cattle	6.60		Foy et al 2002
		Sheep	5.40		Foy et al 2002
		Pig	5.00		Foy et al 2002
		Poultry	5.80		Foy et al 2002
F1.03	Livestock products	Eggs	0.96		McCance and Widdowson 2015
		Milk	1.79		McCance and Widdowson 2015
F2.01	Crop products	Wheat	3.10		AFBI Nutrient Balance 2017 (AFBI pers. comm. 2019)
		Barley	3.40		AFBI Nutrient Balance 2017 (AFBI pers. comm. 2019)
		Oats	2.90		AFBI Nutrient Balance 2017 (AFBI pers. comm. 2019)
		Oilseed rape	14.00		Nutrient Management Guide RB209 (AHDB 2017b)
		Potatoes	0.40		AFBI Nutrient Balance 2017 (AFBI pers. comm. 2019)
		Vegetables	0.23		AFBI Nutrient Balance 2017 (AFBI pers. comm. 2019)
		Fruit	0.22		AFBI Nutrient Balance 2017 (AFBI pers. comm. 2019)
		Mushrooms	0.80		AFBI Nutrient Balance 2017 (AFBI pers. comm. 2019)
F2.02	Fodder crops	Hay	0.59		Nutrient Management Guide RB209 (AHDB 2017b)
		Fresh grass (silage)	1.40		Nutrient Management Guide RB209 (AHDB 2017b)
F3.02 F3.06 F3.08	Animal feed	Average calculated from feed ingredients used	4.60		Feedtables.com (INRA, 2019b) and feedipedia.org (INRA, 2019a)
F3.03 F3.10 F4.01 F4.02 F6.01 F6.02 F6.03	Food both for imports and food waste	Average from selected food products	1.38		McCance and Widdowson 2015

F3.04	Fish landings	Average of reported catch	1.63		McCance and Widdowson 2015
F1.07<br>F1.08<br>F6.03	Livestock waste	Fallen stock and abattoir waste	5.70		Average of live animals from Foy et al 2002
F2.04	Crop and grass farming waste	Average of crop products above	2.70		
F3.10	Meat and fish prep waste sludge	Average of reported values	1.26		Davis and Rudd 1999
F3.11	Dairy waste sludge		0.39		Davis and Rudd 1999
F3.11	Alcohol and beverage waste sludge		0.74		Davis and Rudd 1999
F3.12<br>F3.13	Trade effluent P discharge to water	Average of reported values	0.014		Puchlik and Struk-Sokolowska 2017
F5.02<br>F6.05	Biosolids	Average of reported values	22.3		Nutrient Management Guide RB209 (AHDB 2017b)
F1.05<br>F1.06	Poultry manure for processing and export		7.00		AFBI Pers. Comm. 2019
F1.04	P excretion	Dairy cows		17.1	DEFRA 2006
	P excretion	Beef cows		9.85	DEFRA 2006
	P excretion	All other cattle		8.6	DEFRA 2006
	P excretion	All mature pigs		5.4	DEFRA 2006
	P excretion	Finishers		2.46	DEFRA 2006
	P excretion	Weaners /growers		0.87	DEFRA 2006
	P excretion	Laying birds		0.16	DEFRA 2006
	P excretion	Growing pullets		0.10	DEFRA 2006
	P excretion	Breeding flock		0.16	DEFRA 2006
	P excretion	Broilers		0.08	DEFRA 2006
	P excretion	Turkeys, geese, ducks, others		0.40	DEFRA 2006
	P excretion	All sheep over 1 yr old		1.9	DEFRA 2006
	P excretion	Sheep under 1 yr old		0.045	DEFRA 2006
	P excretion	Horses		4.8	Russell et al 2008

References

AHDB, 2017a. Factors affecting killing-out percentage. https://ahdb.org.uk/knowledge-library/factors-affecting-killing-out-percentage. (Accessed August 2019.

AHDB, 2017b. Nutrient Managament Guide (RB209) Agriculture and Horticulture Development Board.

AHDB, 2018. UK Cattle Yearbook. http://beefandlamb.ahdb.org.uk/wp-content/uploads/2018/10/UK-Cattle-Yearbook-2018.pdf. (Accessed August 2019.

Barry, C.D., McRoberts, C., 2016. Septic tank wastewater discharges in two contrasting catchments: Identification, quantification and distribution of tracers to assess contribution to phosphorus inputs. Unpublished Final Project report to the Department of Agriculture Environment and Rural Affairs.

DAERA, 2018a. Crop and grass areas since 1981. https://www.daera-ni.gov.uk/publications/crop-and-grass-areas-1981-crops-2014. (Accessed Sept 2019.

DAERA, 2018b. Farm animal populations. https://www.daera-ni.gov.uk/publications/farm-animal-population-data. (Accessed August 2019.

DAERA, 2018c. Quantities of the main products in output from 1981 onward. https://www.daera-ni.gov.uk/publications/quantities-main-products-output-1981-onward. (Accessed August 2019.

DAERA, 2018d. Size and Performance of the Northern Ireland Food and Drinks Processing Sector, Subsector Statistics 2017. https://www.daera-ni.gov.uk/sites/default/files/publications/daera/19.20.103%20Size%20and%20Performance%20NI%20Food%20%26%20Drink-revised%20Dec%202019.pdf. (Accessed Sept 2019.

DAERA, 2019a. Animal feed statistics from 2009 - 2019. https://www.daera-ni.gov.uk/publications/animal-feed-statistics-2009-2019. (Accessed August 2019.

DAERA, 2019b. Fertiliser statistics from 2009 - 2019. https://www.daera-ni.gov.uk/publications/fertiliser-statistics-2009-2019. (Accessed August 2019.

DAERA, 2019c. Fish landings into Northern Ireland. https://www.daera-ni.gov.uk/publications/fish-landings-northern-ireland. (Accessed August 2019.

Davis, R., Rudd, C., 1999. Investigation of the criteria for, and guidence on, the landspreading of industrial waste, Environment Agency R&D Technical report P193.

DEFRA, 2006. Nitrogen and phosphorus output of livestock excreta, Final report, Defra project WT0715NVZ.

DEFRA, 2018. United Kindom Slaughter Statistics December 2017. https://assets.publishing.service.gov.uk/government/uploads/system/uploads/attachment_data/file/681620/slaughter-statsnotice-18jan18.pdf. (Accessed Sept 2019.

Forber, K.J., Rothwell, S.A., Metson, G.S., Jarvie, H.P., Withers, P.J.W., 2020. Plant-based diets add to the wastewater phosphorus burden. Environmental Research Letters.

Foy, R.H., Bailey, J.S., Lennox, S.D., 2002. Mineral balances for the use of phosphorus and other nutrients by agriculture in Northern Ireland from 1925 to 2000 - methodology, trends and impacts of losses to water. Irish J. Agr. Food Res. 41(2), 247-263.

HMRC, 2019. Build your own data tables. https://www.uktradeinfo.com/Statistics/BuildYourOwnTables/Pages/Table.aspx. (Accessed Oct 2019.

INRA, 2019a. INRA-CIRAD-AFZ-FAO Feedipedia, Animal feed resources information system. https://www.feedipedia.org/. (Accessed Sept 2019.

INRA, 2019b. INRA-CIRAD-AFZ Feed tables, composition and nutritive values of feeds for cattle, sheep, goats, pigs, poultry, rabbits, horses and salmonids. https://www.feedtables.com/. (Accessed Sept 2019.

McCance, Widdowson, 2015. McCance and Widdowson's The Composition of Foods, sixth ed. The Royal Society of Chemistry, Cambridge.

Naden, P., Bell, V., Carnell, E., Tomlinson, S., Dragosits, U., Chaplow, J., May, L., Tipping, E., 2016. Nutrient fluxes from domestic wastewater: A national-scale historical perspective for the UK 1800-2010. Sci. Total Environ. 572, 1471-1484.

Nesbitt, D., Devine, C., 2018. Northern Ireland Kerbside Waste Composition 2017, summary report.

NISRA, 2018. 2017 Mid Year Population Estimates for Northern Ireland. https://www.nisra.gov.uk/publications/2017-mid-year-population-estimates-northern-ireland. (Accessed Seot 2019.

NIW, 2017. Annual Information Return 2017 for Public Domain. https://www.niwater.com/sitefiles/resources/pdf/reports/air/air17_public_domain_version_v2.pdf. (Accessed Sept 2019.

Puchlik, M., Struk-Sokolowska, J., 2017. Comparison of the composition of wastewater from fruit and vegetables as well as dairy industry, in: Kazmierczak, B., Kutylowska, M., Piekarska, K., TruszZdybek, A. (Eds.), 9th Conference on Interdisciplinary Problems in Environmental Protection and Engineering. E D P Sciences, Cedex A.

Richards, S., Paterson, E., Withers, P.J.A., Stutter, M., 2015. The contribution of household chemicals to environmental discharges via effluents: Combining chemical and behavioural data. J. Environ. Manage. 150, 427-434.

Russell, M.J., Weller, D.E., Jordan, T.E., Sigwart, K.J., Sullivan, K.J., 2008. Net anthropogenic phosphorus inputs: spatial and temporal variability in the Chesapeake Bay region. Biogeochemistry 88(3), 285-304.

Smith, R.V., Jordan, C., Annett, J.A., 2005. A phosphorus budget for Northern Ireland: inputs to inland and coastal waters. J. Hydrol. 304(1-4), 193-202.
